# Circulating Forms of Urokinase-Type Plasminogen Activator Receptor in Plasma Can Predict Recurrence and Survival in Patients with Urothelial Carcinoma of the Bladder

**DOI:** 10.3390/cancers13102377

**Published:** 2021-05-14

**Authors:** Line H. Dohn, Peter Thind, Lisbeth Salling, Henriette Lindberg, Sofie Oersted, Ib J. Christensen, Ole D. Laerum, Martin Illemann, Hans von der Maase, Gunilla Høyer-Hansen, Helle Pappot

**Affiliations:** 1 Department of Oncology, Rigshospitalet, Blegdamsvej 9, 2100 Copenhagen, Denmark; sofie.oersted@regionh.dk (S.O.); hans@vondermaase.dk (H.v.d.M.); helle.pappot@regionh.dk (H.P.); 2 The Finsen Laboratory, Rigshospitalet, Copenhagen Biocenter, Ole Maaloes Vej 5, Building 3, 3rd Floor, 2200 Copenhagen, Denmark; ib.jarle.christensen@regionh.dk (I.J.C.); ole.larum@uib.no (O.D.L.); milleman@its.jnj.com (M.I.); gunillaogbent@hotmail.com (G.H.-H.); 3 Department of Urology, Rigshospitalet, Blegdamsvej 9, 2100 Copenhagen, Denmark; peter.ole.thind@regionh.dk (P.T.); lisbeth.Salling@regionh.dk (L.S.); 4 Department of Oncology, Herlev and Gentofte Hospital, Herlev, Borgmester Ib Juuls Vej 1, 2730 Herlev, Denmark; henriette.lindberg@regionh.dk; 5 Biotech Research and Innovation Centre (BRIC), University of Copenhagen, Ole Maaloes Vej 5, 2200 Copenhagen, Denmark; 6 Department of Surgical Gastroenterology, Hvidovre Hospital, 2650 Hvidovre, Denmark

**Keywords:** bladder cancer, urothelial cancer, blood, plasma, urokinase receptor, uPAR, plasminogen activation, biomarker, survival, recurrence

## Abstract

**Simple Summary:**

Bladder cancer is an aggressive disease and after operation many patients are at risk of recurrence and shortened survival. Specific proteins are known to be of importance in the development of cancers. One of these proteins is the urokinase-type plasminogen activator receptor (uPAR) which exists in different forms. We here investigate the presence of the different uPAR forms in plasma from patients with bladder cancer, and we associate the elevated amount of uPAR forms with survival. We find that high levels of all uPAR forms is associated with short survival in patients with bladder cancer and suggest that this in the future might help improve handling of the disease.

**Abstract:**

Urothelial carcinoma of the bladder is a highly aggressive disease characterised by a very heterogeneous clinical outcome. Despite cystectomy, patients still have a high recurrence risk and shortened survival. Urokinase-type plasminogen activator receptor (uPAR) is present in tumour tissue specimens from patients with urothelial carcinoma. The different uPAR forms in blood are strong prognostic markers in other cancer types. We investigate the presence of different uPAR forms in tumour tissue and test the hypothesis that preoperative plasma levels of the uPAR forms predict recurrence free survival, cancer specific survival, and overall survival in patients treated with cystectomy for urothelial carcinoma. Using Western blotting we analyse neoplasia and adjacent benign-appearing urothelium from randomly selected patients for the presence of intact and cleaved uPAR forms. Prospectively collected preoperative plasma samples from 107 patients who underwent radical cystectomy for urothelial carcinoma are analysed. The different uPAR forms are measured by time-resolved fluorescence immunoassays. uPAR in tumour tissue from patients with urothelial carcinoma is demonstrated in both an intact and cleaved form. The different uPAR forms in plasma are all significantly associated with both recurrence free survival, cancer specific survival, and overall survival, high concentrations predicting short survival. uPAR (I) has the strongest association with a HR of 2.56 for overall survival. In the multivariable survival analysis uPAR (I) is significantly associated with cancer specific survival and overall survival.

## 1. Introduction

For patients with high-risk non-muscle invasive and muscle invasive urothelial carcinoma of the bladder (UCB) without radiological signs of metastasis, radical cystectomy (RC) with lymphadenectomy is the standard, potentially curative, treatment [1,2]. RC affords effective local disease control; however, around 50% of patients with node negative disease will die of their cancer within five years [3,4]. Patients with histologically confirmed lymph node metastases have an even higher risk [3]. Neoadjuvant cisplatin-based chemotherapy is recommended in patients with muscle invasive disease without signs of lymph node metastases or distant metastasis, as it has shown an 8% absolute improvement in survival at five years [2,5]. Despite aggressive therapy, patients suffer from high rates of disease recurrence and shortened survival. Current tools, such as standard histopathologic risk factors and imaging are insufficient to detect micro-metastatic disease [6,7]. Hence, there is a strong need for additional biomarkers that at an early time-point can help capture the true clinical and biological potential of a tumour to permit a more individualised guidance for therapy or follow-up. 

Detachments of cancer cells from the primary tumour, cancer cell migration and invasion into the surrounding tissue require degradation and remodelling of the extracellular matrix, which is mediated by a complex array of extracellular proteases [8]. The plasminogen activation system is one of the central protease systems involved in these processes. uPAR localises its ligand urokinase-type plasminogen activator (uPA) to the cell surface. Full length uPAR [uPAR (I−III)] consists of three domains and is attached to the cell membrane by a glycolipid anchor on domain III [9]. uPA bound to uPAR is capable of cleaving neighbouring cell-bound uPAR, liberating the uPAR (I) and leaving the cleaved form, uPAR (II−III), on the cell surface [9]. Therefore, the cleaved uPAR forms reflect the activity of the plasminogen activation system. uPAR (I−III) and uPAR (II−III) can be shed from the cell surface; consequently one intact and two cleaved uPAR forms are present in blood. Use of immunoassays measuring the individual forms of uPAR have demonstrated that the cleaved uPAR forms are strong prognostic biomarkers in other cancers, and better than the total amount of uPAR [10,11,12]. The cleaved uPAR forms measured in blood have not been investigated in UCB but we know from studies in tumour tissue from patients with UCB that a significant association exists between uPAR expression and increasing tumour stage, as well as an association between uPAR expression and poor survival [13,14,15]. The uPAR antibodies that work on paraffin embedded tissue are, however, not able to distinguish between different uPAR forms [9].

Taken all together there is an ongoing need to identify new selection markers which can be important tools to detect micro-metastatic disease—the earliest form of advanced bladder cancer. The uPAR forms measured in blood might be such new selection markers.

The aim of the study was to identify the presence of different uPAR forms in tissue and blood from UCB patients. We have used Western blotting of tumour tissue and time-resolved fluorescence immunoassays on plasma samples. In addition, we have tested the hypothesis that high preoperative plasma levels of the intact and cleaved uPAR forms can predict recurrence free survival, cancer specific survival, and overall survival in patients treated with RC for UCB.

## 2. Materials and Methods

### 2.1. Tumour Tissue

For Western blotting, fresh frozen muscle invasive tumour tissue as well as adjacent benign-appearing tissue from two patients—not included in the blood sample material below—treated with RC for UCB was analysed. 

### 2.2. Patients and Blood Samples

Prospectively collected, preoperative EDTA plasma levels of the different uPAR forms were measured in 107 patients (20 women [19%] and 87 men [81%]; median age 67 years, range 44–81 at treatment start), who underwent RC with intended extended lymphadenectomy between March 2012 and April 2015 at the Department of Urology, Rigshospitalet, Copenhagen or the Department of Urology, Herlev Hospital, Denmark. The median number of lymph nodes resected was 18 (range 4–44). Six patients had ≤10 lymph nodes removed. Eligible for inclusion were individuals with histopathological diagnosis of UCB with no evidence of other cancer within the last five years. Indications for RC were high-risk non-muscle invasive or muscle invasive disease without signs of lymph node metastases or distant metastasis, as investigated by computed tomography (CT) scan. Patients with T2–T4a were candidates for neoadjuvant chemotherapy. This supplementary treatment was, however, not applied for all study patients, as neoadjuvant chemotherapy was under implementation during the study period. 

### 2.3. Histopathology

Histological features were collected from the histological reports. Pathologic tumour stage and grade were achieved using criteria from the UICC [16] and the WHO grading system [17]. Pathologic subgroups were defined as pT ≤ pT2, pN0 vs. pT ≥ pT3, pN0 vs. pTany, pN+.

### 2.4. Postoperative Follow-Up

Follow-up schedule was performed according to institutional protocols. In general, patients with organ confined node negative disease (pT ≤ pT2, pN0) were CT-scanned 6, 12 and 24 months postoperatively, while patients with non-organ confined disease (pT ≥ pT3, pN0 or pTany, pN+) were CT-scanned 6, 12, 18, 24 and 36 months after surgery.

Follow-up was defined as the interval from RC until death; if death had not occurred, data was censored at time of analysis (August 2017). The primary endpoint of this study was recurrence free survival (RFS) and the secondary endpoints were cancer specific survival (CSS) and overall survival (OS) [18]. Date of first recurrence, as well as date and cause of death, were obtained by retrospective chart review. Cancer detection in the ureter or urethra was coded as a second primary cancer and not as local or distant recurrence. Perioperative mortality (any death within 30 days of surgery) was censored at time of death for CSS analysis [18].

### 2.5. Blood Samples

Blood samples were collected prior to treatment start defined as the date of cystectomy or in patients receiving neoadjuvant chemotherapy as day 1 of chemotherapy. For preparation of EDTA plasma, blood samples were collected and handled as previously described [19]. 

### 2.6. Measurements of uPAR Forms in Plasma

Three different time-resolved fluorescence immunoassays (TR-FIAs) were used: TR-FIA 1 measuring uPAR (I−III) [20], TR-FIA 2 measuring uPAR (I−III) + uPAR (II−III) [20], and TR-FIA 4 measuring uPAR (I) [10]. All three assays have been validated for use in 20% EDTA plasma [19,21]. Samples were measured in duplicate, and the mean values were used for statistical analyses. The inter-assay variability was below 10 CV%. The CV% for the duplicate measurements was below 20. The limit of quantification in 20% EDTA plasma is for TR-FIA 1 0.8 pmol/L, TR-FIA 2 2.3 pmol/L [21] and for TR-FIA 4 0.41 pmol/L [19]. The recovery of uPAR (I−III) in 20% EDTA plasma is 91% for TR-FIA 1, 95% for TR-FIA 2 [21], and the recovery of uPAR (I) is 91% for TR-FIA 4 [19]. 

### 2.7. Western Blot

Frozen tissue was pulverised using a dry ice cooled tissue homogeniser. The tissue powder preparations were lysed using temperature induced phase separation in Triton X-114 containing buffer (0.1 M Tris/HCl, pH 8.1, 1% Triton X-114, 10 mM EDTA, 10 µg/mL aprotinin and 1 mM phenyl-methyl-sulfonyl fluoride; 10 mL buffer/g frozen tissue) [22]. uPAR (I−III) and uPAR (II−III) separate to the detergent-phase because of their glycolipid anchor. The detergent-phases were chemically reduced, de-glycosylated using *N*-glycosidase F (Roche Diagnostic, Mannheim, Germany) and analysed by Western blotting using the monoclonal antibody S1 [23]. Detergent-phase from 2.5 mg tissue was loaded in each lane. Detergent-phase from 5 × 10^5^ cells of the human histiocytic lymphoma cell line U937 was loaded as control [23]. The proteins were separated on 4–12% SDS-PAGE and were electroblotted onto polyvinylidene difluoride membranes using the iBlot system (Life Technologies) and Western blotting conducted essentially as described [24] using 2 μg/mL of S1 and 1.3 μg/mL horseradish peroxidase conjugated rabbit anti-mouse IgG (P0161, Dako, Glostrup, Denmark). 

### 2.8. Statistics

Descriptive statistics for continuous covariates are presented by the median as well as the minimum and maximum, and categorical variables by the frequencies. Spearman rank correlation was used as a measure of association between the uPAR levels and tests comparing levels between categories were done using the Wilcoxon rank sum test. Analyses of time to event data (RFS, CSS and OS) were performed using the Cox proportional hazards model. Results are presented by hazard ratios (HR) with 95% confidence intervals (CI). Final multivariable models were identified using backwards selection. The concordance index as a measure of discrimination was calculated for each outcome [25]. The Cox models have been assessed based on martingale residuals. The levels of the different uPAR forms have been evaluated by the actual score on the log scale (base 2, implying that HRs are for a two-fold difference in levels) and dichotomised based on the 95th percentile from a reference set [19]. The level of significance was set to 5%. All statistical calculations have been done using SAS (v9.4, SAS Institute, Cary, NC, USA) and R (R version 3.6.3).

## 3. Results

### 3.1. Intact and Cleaved uPAR in UCB Tumour Tissue

**Figure 1:**
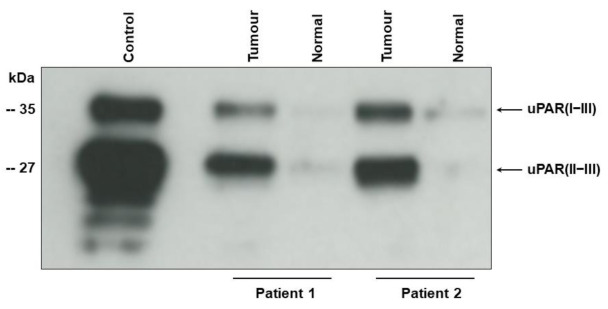
Intact and cleaved uPAR in tumour tissue from two patients with UCB.

Western blot of tumour tissue samples shows two bands corresponding to the intact (uPAR (I−III)) (35 kDa), and cleaved uPAR (uPAR (II−III)) (27 kD) [9,23] (Figure 1). For the original Western blot figure see Figures S1 and S2). In agreement with our previous findings using immunohistochemistry [13,14] the uPAR forms are highly up-regulated in the tumour tissue as compared to the adjacent benign-appearing tissue. 

Detergent-phase of UCB tissue (tumour) and adjacent benign appearing tissue (normal) was subjected to enzymatic de-glycosylation before electrophoresis to allow complete separation of uPAR (I−III) and uPAR (II−III). The monoclonal antibody S1 reacting with an epitope on domain II and III of chemically reduced uPAR [23] was used for Western blotting. Detergent-phase from U937 cells (control) was applied as positive control [23]. Electrophoretic mobility of standard proteins is indicated to the leftt. 

### 3.2. Association of Preoperative Plasma uPAR Forms with Clinical and Pathologic Characteristics

The uPAR forms were measured in EDTA plasma samples from 107 patients. Patient characteristics are presented in Table 1. 

uPAR (I−III) was significantly associated with gender, uPAR (I−III) + uPAR (II−III) was significantly associated with gender, pathologic stage and resection margin, whereas uPAR(I) was significantly associated with gender and lymph node metastases. No association was found between plasma levels of any uPAR form and age, tumour grade, concomitant carcinoma in situ (CIS) or lymph vascular invasion. Correlations (rs) between uPAR (I−III) and uPAR (I−III) + uPAR (II−III), uPAR (I−III) and uPAR (I), and uPAR (I−III) + uPAR (II−III) and uPAR (I) were 0.74, 0.44 and 0.69, respectively. Association of the preoperative plasma uPAR forms with clinical and pathological characteristics are presented in Table 2.

### 3.3. Association of Preoperative Plasma uPAR Forms with Clinical Outcome

The median follow-up was 34 months, range 20–55 months. During this time 25 (23%) patients experienced disease recurrence and 28 (26%) patients had died, hereof 21 (75%) of UCB. No patients died perioperatively. 

The different uPAR forms were all significantly associated with both RFS, CSS and OS, with uPAR (I) having the strongest association—for RFS a HR of 2.26; CI (95%): 1.45–3.50; *p* = 0.0003, for CSS a HR of 2.63; CI (95%): 1.58–4.38; *p* = 0.0002, and for OS a HR of 2.56; CI (95%): 1.64–4.00; *p* = 0.0001 were demonstrated, i.e., with high concentrations predicting short survival. Additionally, we found tumour stage, lymph node status, vascular invasion, and positive resection margin statistically significantly associated with RFS, CSS, and OS, as well as a significant association between female gender and worse CSS and OS. No association was seen between clinical outcome and age, tumour grade, and neoadjuvant chemotherapy. The univariate analyses are presented in Table 3. 

### 3.4. Multivariable Survival Analysis

In the reduced multivariable survival analysis, uPAR (I) was significantly associated with CSS and OS. Tumour stage, vascular invasion, and uPAR (I−III) + uPAR (II−III) were significantly associated with RFS (Table 4). Multivariate analyses for recurrence using uPAR (I−III) + uPAR (II−III) demonstrated HR = 7.55 (95% CI: 2.03–28.03, *p* = 0.003), for CSS using uPAR (I) HR = 2.12 (95% CI: 1.28–3.51, *p* = 0.004), and for OS using uPAR(I) HR = 2.11 (95% CI: 1.35–3.31, *p* = 0.001). Concordance index (C-index) as a measure of discrimination was calculated for each outcome (Table 4).

### 3.5. Application of Previous Determined Reference Intervals of the Different uPAR Forms

In a reference material consisting of interpreted healthy individuals, the 95th percentile upper limits of the reference intervals of the different uPAR forms in EDTA plasma were determined [19]. We know from the reference material that the uPAR levels are dependent on age and gender.

**Figure 2:**
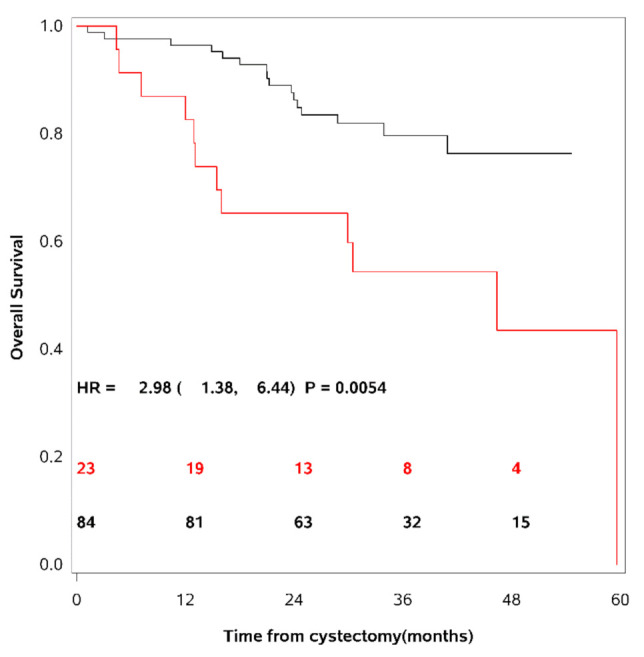
Overall survival as a function of soluble uPAR (I−III) + uPAR (II−III).

These 95th percentile upper limits were used as cut-points to dichotomise the patient cohort into patients having elevated levels of the different uPAR forms as compared to others. 36% of the UCB patients had elevated levels of uPAR (I−III), 24% had elevated levels of uPAR (I−III) + uPAR (II−III), and 27% had elevated levels of uPAR (I). As mentioned above, the actual cut-point for each marker is dependent on age and gender [19]. Univariate survival analyses demonstrated a decrease in RFS, CSS and OS in patients with levels above this cut-point of any of the uPAR forms. The strongest association was seen between uPAR (I−III) + uPAR (II−III) and OS (HR = 2.98; CI (95%): 1.38–6.44; *p* = 0.0054) (see Table 3). Figure 2 show OS as a function of soluble uPAR (I−III) + uPAR (II−III).

Multivariate analyses for recurrence using the dichotomised uPAR (I−III) + uPAR (II−III) level demonstrated HR = 3.59 (95% CI: 1.36–9.50, *p* = 0.01), for CSS using the dichotomised uPAR (I) level HR = 2.15 (95% CI: 0.81–5.69, *p* = 0.12), and for OS using the dichotomised uPAR (I) level HR = 2.78 (95% CI: 1.22–6.33, *p* = 0.015). 

Kaplan-Meier survival curves are shown using the 95th percentile of the reference intervals for dichotomisation of the patient cohort. The red line = patients with uPAR above the cut-point, the black line = patients with uPAR levels below the cut-point. The number of patients at risk of death is shown for time of inclusion, 12, 24, 36, and 48 months after cystectomy. A strong association was seen between patients with levels of uPAR (I−III) + uPAR (II−III) above the cut-point and a decrease in overall survival. 

## 4. Discussion

This is the first report demonstrating a significant association between high preoperative levels of the different forms of uPAR in plasma and shortened RFS, CSS and OS in patients undergoing RC for UCB. In the univariate survival analyses, the strongest association with both RFS, CSS and OS were seen for uPAR (I). In the reduced multivariate survival analysis, uPAR (I) was significantly associated with CSS and OS. Thus, the prognostic impact of uPAR (I) was independent of known prognostic factors such as tumour stage and lymph node status. This study was performed in a period where neoadjuvant chemotherapy was under implementation and had not become a routine, which explains the relatively small fraction of patients in our cohort having received this treatment. The small number of patients receiving neoadjuvant chemotherapy most likely explains that no association was seen between clinical outcome and this treatment modality. 

As a control population we used the reference levels determined by Thurison et al. [19]. We chose the 95th percentile of the previous established reference intervals as cut-points to dichotomise our patient cohort. This revealed a decrease in RFS, CSS and OS in patients with levels above this cut-point of any uPAR form. In this setting, the strongest association to clinical outcome was seen for uPAR (I−III) + uPAR (II−III). Using the same approach in patients with colorectal cancer, uPAR (I) had the strongest discriminatory power [19]. Results may be influenced by the cut-off chosen to separate groups. These differences between cancer types could also reflect different levels of activation of the plasminogen activation system as well as of other proteases. 

Interestingly, using an assay measuring the collective amounts of uPAR forms, Shariat et al. previously showed no significant impact of preoperative plasma levels of uPAR on survival in a small cohort of patients treated with RC for UCB [26]. The different methods used for quantification of the plasma uPAR levels in the study by Shariat et al. and in our study is most likely the reason for the diverse results. Shariat et al. do not present the antibodies employed in their ELISA and reveal no details on which uPAR forms their ELISA measures. We used only fully characterised monoclonal antibodies in our TR-FIAs, measuring specific uPAR forms. Cleavage of uPAR (I−III) is a sign of increased protease activity and therefore the cleaved forms, in particular uPAR (I), are stronger associated to overall survival than a mixture of the different uPAR forms, most likely measured by the ELISA used in the study by Shariat et al.

In addition to identifying the three uPAR forms in plasma and their association to clinical outcome, we demonstrated that both intact [uPAR (I−III)], and cleaved [uPAR(II−III)] forms of uPAR are present in urothelial carcinoma tissue. The purpose of this Western blot experiment was not to compare the quantities of uPAR (I−III) and uPAR (II−III) in the individual sample but to show that they are present and that the levels are higher in the tumour tissue than in the adjacent benign-appearing tissue. Because uPAR (I) does not have a glycolipid anchor, it is soluble and never found in detergent extracts of membrane proteins (tissue lysates) in contrast to uPAR (I−III) and uPAR (II−III), which is attached to the cell surface by a glycolipid anchor on domain III. uPAR (I−III) is cleaved on the cell surface by uPA in the linker region between domain I and II, liberating uPAR (I) and leaving uPAR (II−III) attached to the cell surface. We have previously published the entire blots with all the molecular weight markers (see Figures 1–3 in reference [23]. This experiment was done to ensure that cleaved uPAR might originate from the tumour tissue and not to demonstrate any correlations. This is in accordance with our previous findings in breast cancer [27]. 

Various sources of uPAR can contribute to the levels found in blood. It is widely accepted that increased local production of uPAR at the primary tumour site is a marker of high invasive potential which can be directly translated into poor prognosis [13,14,28,29]. Although no direct correlation between tumour tissue content and blood levels of uPAR has been reported [30], it is believed that the enhanced levels of circulating uPAR forms in cancer patients compared with healthy individuals [19] are derived predominantly from tumour tissue and accompanying stromal reactions, such as inflammation [21,31]. The concentration of the different uPAR forms in plasma is low. We have previously immunoaffinity purified soluble full-length uPAR (uPAR (I−III)) using a monoclonal antibody against an epitope on domain I [32]. This was done to visualise uPAR (I) by Western blotting in plasma from healthy individuals. We were, however, not able to visualise any uPAR (I) by Western blotting, most likely because the sensitivity of a Western blot is much lower than that of an immunoassay, including TR-FIA 4. 

The present findings and our previous findings by immunohistochemistry [13,14] support the hypothesis that the tumour tissue might be the origin of the enhanced plasma levels of uPAR in patients with UCB. We did not measure the levels of the different uPAR forms in tumour tissue lysates from all patients with UCB for several reasons. The pathologist must have priority in selection of tumour tissue for diagnosis and, additionally, collection of fresh frozen tumour tissue is not standard clinical practice. Furthermore, tumour tissue is rather heterogeneous and the piece available for extraction might not be representative. Peripheral blood on the contrary is considerably more homogeneous and easier to collect than tumour tissue. Lastly, it was previously shown in breast cancer that there was no correlation between the levels found in tumour tissue and blood from the same patient [30].

Intense investigation of molecular alterations involved in different steps of invasion, progression, and metastases of UCB has revealed various promising tissue-, blood, and urine-based biomarkers as predictors for outcome in different bladder cancer settings [33,34,35,36,37,38]. Several predictive and prognostic models have been designed, but to this point none of these have reached a sufficient level of discrimination to allow implementation in daily clinical practice [37,39,40,41,42,43]. 

Owing to the complexity of the molecular abnormalities in UCB, it is unlikely that a single marker can accurately segregate tumours of similar clinicopathologic phenotypes into precise prognostic categories. To improve clinical outcome in patients with muscle invasive UCB we believe, as do other researchers, that a combination of known clinicopathological risk factors and independent, complementary molecular biomarkers might be most valuable for identification of those patients who are at highest risk of experiencing a recurrence after radical cystectomy, and therefore should be offered additional systemic therapy, or should be offered palliative treatment to avoid morbidity after radical cystectomy. The field is evolving rapidly, and the combination of possible selection markers should be based on scientific evidence, technical feasibility, and clinical relevance. At present there are, however, no suggested standard risk markers [44].

Our findings indicate that preoperative plasma levels of the cleaved uPAR forms could have a possible role in identification of patients at high risk of disease recurrence and cancer specific death which might allow a more individualised patient counselling and more focused use of perioperative therapy and/or a more individualised follow-up schedule. The patient population is, however, too small to draw firm conclusions and our data need to be validated in a larger independent patient cohort. Thus, the clinical implications are not yet clarified. 

Additionally, it could be interesting to investigate the potential of soluble uPAR as a marker of recurrence during follow-up and/or as an indicator of effectiveness of ongoing treatment. If soluble uPAR is effective for these purposes, this might help to deliver more timely and relevant systemic treatment when the disease becomes advanced. 

## 5. Conclusions

Our study is the first to demonstrate a significantly association between high preoperative levels of the different uPAR forms in plasma, particularly the liberated domain I—uPAR (I)—and recurrence as well as survival in patients with urothelial neoplasia undergoing radical cystectomy. 

## Figures and Tables

**Table 1:** Patient characteristics (*n* = 107).

Characteristics	*N* (%)
Age	
Years median (range)	67 (44–81)
Gender	
Female	20 (19)
Male	87 (81)
Pathologic stage (cystectomy specimen)	
T0	36 (34)
CIS	15 (14)
Ta	4 (4)
T1	4 (4)
T2	22 (20, 5)
T3	22 (20, 5)
T4	4 (4)
Pathologic stage (cystectomy specimen)	
pT ≤ pT2 pN0	76 (71)
pT ≥ pT3 pN0	17 (16)
pTany pN+	14 (13)
Pathologic grade (cystectomy specimen)	
LG	2 (2)
HG	69 (64)
Concomitant CIS ^1^	
No	65 (61)
Yes	42 (39)
Lymph vascular invasion	
No	93 (87)
Yes	14 (13)
Resection margin	
No	101 (94)
Yes	6 (6)
Lymph node metastasis	
No	93 (87)
Yes	14 (13)
Neoadjuvant chemotherapy	
No	79 (74)
Yes	28 (26)

^1^ CIS = carcinoma in situ; LG = low grade; HG = high grade.

**Table 2:** Association of the preoperative plasma uPAR forms with clinical and pathological characteristics.

Characteristics	uPAR (I−III)		uPAR (I−III) + uPAR (II−III)		uPAR (I)	
	pmol/L		pmol/L		pmol/L	
	Median (Min-Max)	*p*-Value	Median (Min-Max)	*p*-Value	Median (Min-Max)	*p*-Value
Age						
67 (44–81) ^a^	0.15	0.13	0.13	0.17	−0.03	0.78
Gender						
Female	53.65 (31.98–91.43)		94.73 (68.90–226.21)		38.85 (17.04–754.88)	
Male	42.69 (19.34–76.66)	0.003	84.68 (51.60–156.67)	0.02	35.60 (16.40–124.15)	0.05
Pathologic stage (cystectomy specimen) ^b^						
pT ≤ pT2 pN0	42.72 (19.34–76.66)		85.14 (51.60–156.67)		34.09 (16.40–124.15)	
pT ≥ pT3 pN0	53.28 (31.44–68.37)	0.15	101.25 (73.52–145.19)	0.03	42.99 (17.04–86.66)	0.06
pTany pN+	42.25 (27.47–91.43)		84.99 (55.20–226.21)		34.00 (21.08–754.88)	
Pathologic grade						
LG	54.96 (45.91–64.00)		107.33 (79.36–135.29)		58.14 (57.62–58.65)	
HG	44.50 (25.62–91.43)	0.59	87.51 (51.60–226.21)	0.71	36.45 (16.40–754.88)	0.38
Concomitant CIS						
No	42.71 (19.34–91.43)		88.19 (53.07–226.21)		35.91 (19.38–754.88)	
Yes	48.09 (26.49–76.66)	0.39	89.56 (51.60–132.43)	0.96	38.89 (16.40–142.10)	0.44
Lymph vascular invasion						
No	44.05 (19.34–91.43)		88.19 (51.60–156.67)		35.91 (16.40–754.88)	
Yes	46.06 (32.02–78.11)	0.53	89.15 (57.15–226.21)	0.31	39.60 (24.68–142.10)	0.20
Resection margin						
Negative	43.23 (19.34–91.43)		87.51 (51.60–156.67)		35.91 (16.40–754.88)	
Positive	51.12 (44.41–78.11)	0.09	128.56 (77.47–226.21)	0.05	46.75 (25.00–107.60)	0.27
Lymph node metastasis						
No	44.41 (19.34–76.66)		89.35 (51.60–156.67)		36.45 (16.40–124.15)	
Yes	42.25 (27.47–91.43)	0.92	84.99 (55.20–226.21)	0.87	34.00 (21.08–754.88)	0.05
Neoadjuvant chemotherapy						
No	44.41(/19.34–78.11)		87.51 (5160–228.21)		35.60 (16.40–142.10)	
Yes	43.55 (25.62–91.43)	0.85	89.42 (57.04–156.67)	0.97	37.61 (19.38–754.88)	0.17

Descriptive statistics presented with median, minimum and maximum concentrations for the different uPAR forms (pmol/L). ^a^ Spearman’s rank correlation coefficient (rs), *p*-value testing the hypothesis that rs = 0. ^b^ *p*-values are for the Wilcoxon rank sum test comparing the levels of the uPAR concentrations for baseline patients characteristic. CIS = carcinoma in situ; LG = low grade; HG = high grade.

**Table 3:** Association of the preoperative plasma uPAR forms with clinical outcome (univariate analyses).

Patient Characteristics	RFS ^a^			CSS ^a^			OS ^a^		
	HR	95% CI	*p*-Value	HR	95% CI	*p*-Value	HR	95% CI	*p*-Value
uPAR levels log transformed base 2									
uPAR (I−III)	3.11	1.03–9.44	0.045	3.41	1.00–11.67	0.051	3.85	1.26–11.72	0.018
uPAR (I−III) + uPAR (II−III)	4.20	1.39–12.67	0.011	4.03	1.37–11.87	0.012	4.95	1.90–12.92	0.001
uPAR (I)	2.26	1.45–3.50	0.0003	2.63	1.58–4.38	0.0002	2.56	1.64–4.00	0.0001
uPAR levels dichotomised according to reference intervals ^b^									
uPAR (I−III)	1.90	0.87–4.17	0.109	1.74	0.74–4.09	0.207	1.97	0.93–4.20	0.078
uPAR (I−III) + uPAR (II−III)	2.02	0.89–4.56	0.093	2.19	0.91–5.30	0.081	2.93	1.37–6.27	0.006
uPAR (I)	1.62	0.74–3.56	0.226	1.57	0.67–3.69	0.303	1,84	0.86–3.91	0.115
Clinicopathological characteristics									
Age pr. 10 yr. age diff.	1.00	0.63–1.60	1.000	0.87	0.53–1.44	0.594	0.98	0.62–1.54	0.922
Gender									
Female vs. Male	3.60	1.26–8.00	1.00	3.73	1.57–8.85	0.003	2.35	1.05–5.24	0.038
Tumor stage (cystectomy specimen)									
pT ≥ pT3 pN0 vs. pT ≤ pT2 pN0	8.07	2.79–23.31	0.0001	9.00	2.94–27.58	0.0001	5.30	2.15–13.10	0.0003
pTany pN+ vs. pT ≤ pT2 pN0	17.51	6.38–48.05	<0.0001	11.71	3.08–35.93	<0.0001	6.32	2.48–16.12	0.0001
Lymph node metastasis									
Yes vs. No	8.62	3.85–19.23	0.0001	5.26	2.17–12.66	0.0002	3.85	1.67–8.85	0.0015
Pathologic grade									
HG vs. LG	NA ^c^		1.00	NA ^c^		1.00	NA ^c^		0.99
Vascular invasion									
Yes vs. No	6.99	3.09–15.87	0.0001	5.20	2.15–12.66	0.0003	3.86	1.68–8.93	0.002
Resection margin									
Positive vs. Negative	6.80	2.54–18.18	0.0001	5.13	1.72–15.38	0.003	3.65	1.26–10.64	0.017
Neoadjuvant chemotherapy									
Yes vs. No	0.71	0.27–1.89	0.49	0.95	0.35–2.61	0.92	1.41	0.61–3.26	0.42

^a^ Analyses have been done using the Cox proportional hazards model, and the results are presented by the HR with 95% CI. The reference levels are those with an expected good prognosis, but for neoadjuvant therapy, the reference is those not receiving neoadjuvant therapy. ^b^ Patients were dichotomized using the 95th percentile upper limit of the previous determined reference intervals for the different uPAR forms. ^c^ NA: not accessible because of the limited number of patients with uPAR negative combined with the low event rate. LG = low grade; HG = high grade.

**Table 4:** Reduced multivariate analyses.

Patient Characteristics	RFS (C-Index = 0.83)			CSS (C-Index = 0.81)			OS (C-Index = 0.78)		
	HR	95% CI	*p*-Value	HR	95% CI	*p*-Value	HR	95% CI	*p*-Value
Clinicopathological characteristics									
pT ≥ pT3 pN0 vs. pT ≤ pT2 pN0	4.5	1.51–13.84	0.007	7.8	2.56–24.19	0.0003	4.55	1.84–11.26	0.001
pTany pN+ vs. pT ≤ pT2 pN0	19.54	6.00–63.61	<0.0001	11.06	3.50–34.87	<0.0001	5.70	2.15–15.13	0.0005
Vascular invasion	2.56	1.02–6.25	0.045						
uPAR (I−III) + uPAR (II−III)	7.55	2.03–28.03	0.003						
uPAR (I)				2.12	1.28–3.51	0.004	2.11	1.35–3.31	0.001

## Data Availability

The data that support the findings of this study are available from corresponding author Line Hammer Dohn but restrictions apply to the availability of these data, which were used under license for the current study, and so are not publicly available. According to Danish law data cannot be transferred to other institutions without a data transfer agreement.

## Supplementary Materials

The following are available online at https://www.mdpi.com/article/10.3390/cancers13102377/s1, Figure S1: original Western blot figure of Figure 1.
