# Supplementary figures and images for: Circulating Forms of Urokinase-Type Plasminogen Activator Receptor in Plasma Can Predict Recurrence and Survival in Patients with Urothelial Carcinoma of the Bladder
Source: Cancers (Basel). 2021 May 14;13(10):2377. doi: 10.3390/cancers13102377 (PMC8156453; doi:10.3390/cancers13102377)

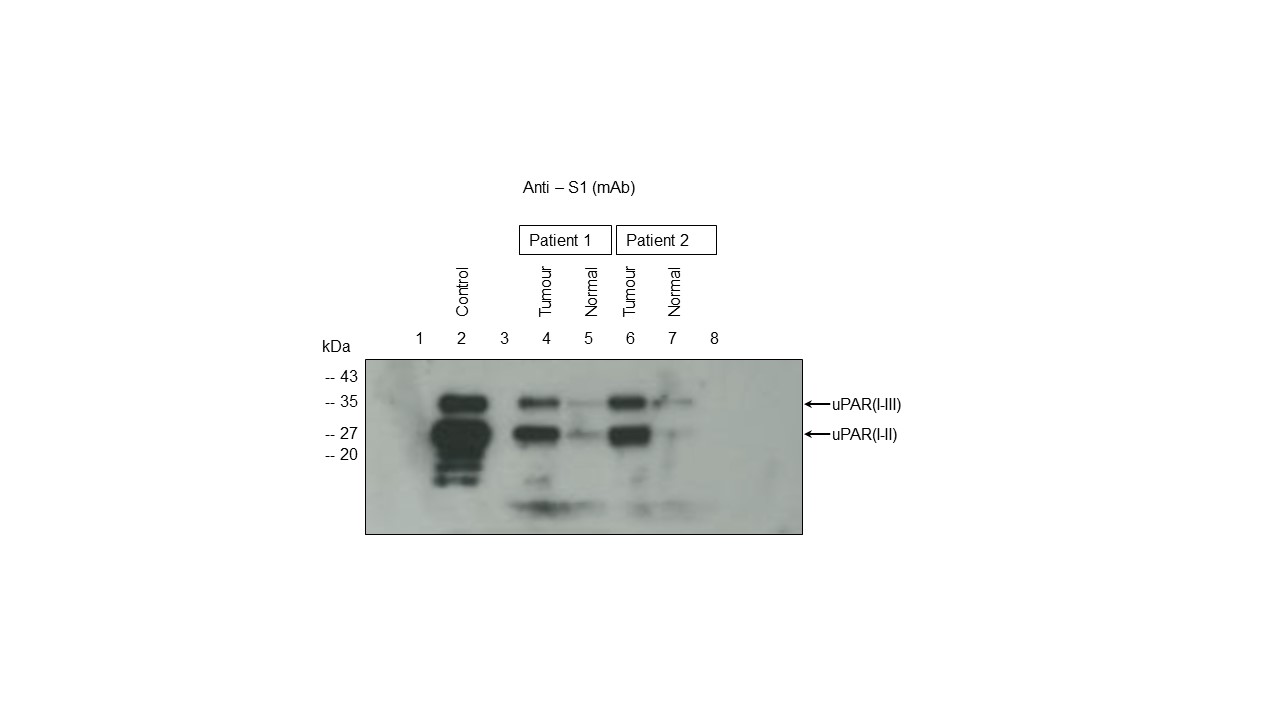

Supplement: Supplementary file 1 [file cancers-13-02377-s001.zip › Figure S1 (modified).jpg]

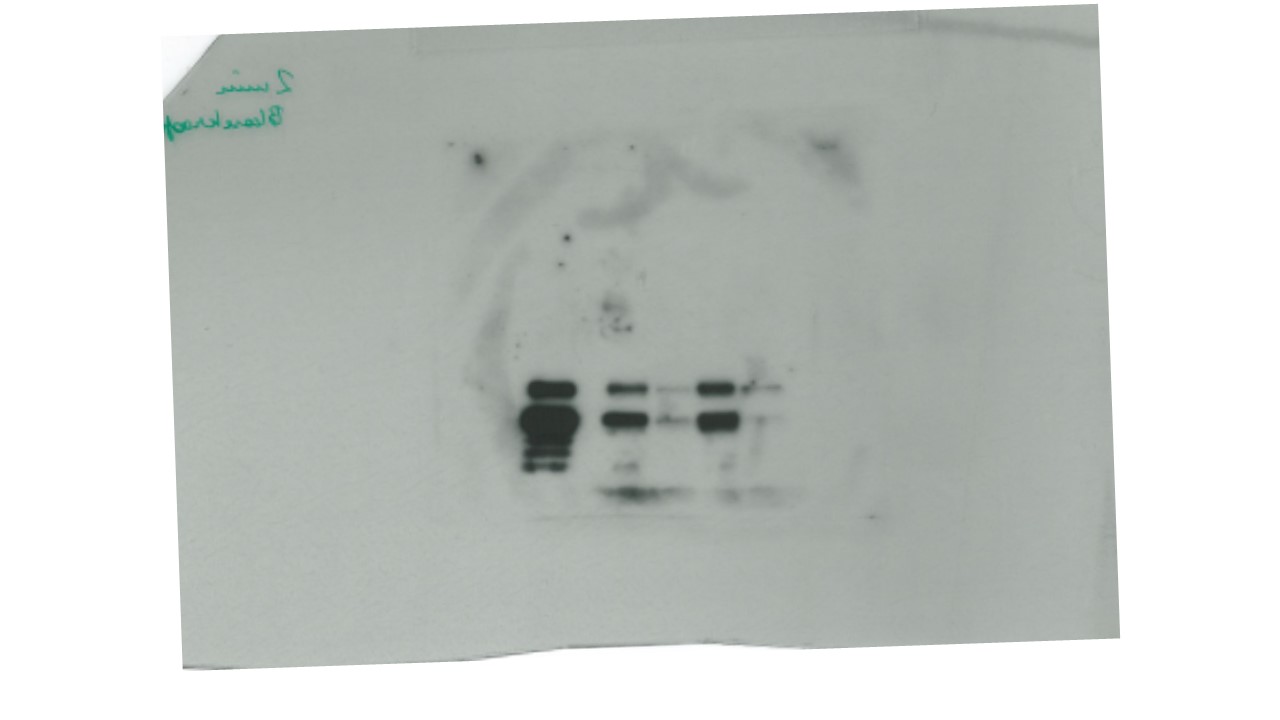

Supplement: Supplementary file 1 [file cancers-13-02377-s001.zip › Figure S1 (scan).jpg]
